# Sex-based *de novo* transcriptome assemblies of the parasitoid wasp *Encarsia suzannae*, a host of the manipulative heritable symbiont *Cardinium hertigii*

**DOI:** 10.46471/gigabyte.68

**Published:** 2022-09-02

**Authors:** Dylan L. Schultz, Evelyne Selberherr, Corinne M. Stouthamer, Matthew R. Doremus, Suzanne E. Kelly, Martha S. Hunter, Stephan Schmitz-Esser

**Affiliations:** ^1^ Department of Animal Science, Iowa State University, Ames, IA 50011, USA; ^2^ Interdepartmental Microbiology Graduate Program, Iowa State University, Ames, IA 50011, USA; ^3^ Unit of Food Microbiology, Institute of Food Safety, Food Technology and Veterinary Public Health, Department for Farm Animals and Veterinary Public Health, University of Veterinary Medicine Vienna, 1210 Vienna, Austria; ^4^ Department of Entomology, The University of Arizona, Tucson, AZ 85721, USA

## Abstract

Parasitoid wasps in the genus *Encarsia* are commonly used as biological pest control agents of whiteflies and armored scale insects in greenhouses or the field. They are also hosts of the bacterial endosymbiont *Cardinium hertigii*, which can cause reproductive manipulation phenotypes, including parthenogenesis, feminization, and cytoplasmic incompatibility (the last is mainly studied in *Encarsia suzannae*). Despite their biological and economic importance, there are no published *Encarsia* genomes and only one public transcriptome. Here, we applied a mapping-and-removal approach to eliminate known contaminants from previously-obtained Illumina sequencing data. We generated *de novo* transcriptome assemblies for both female and male *E. suzannae* which contain 45,986 and 54,762 final coding sequences, respectively. Benchmarking Single-Copy Orthologs results indicate both assemblies are highly complete. Preliminary analyses revealed the presence of homologs of sex-determination genes characterized in other insects and putative venom proteins. Our male and female transcriptomes will be valuable tools to better understand the biology of *Encarsia* and their evolutionary relatives, particularly in studies involving insects of only one sex.

## Background

*Encarsia suzannae* are minute parasitoid wasps within the order Hymenoptera. Our interest in this species is due to their unusual behavior and biology, their use as a biological control of the important whitefly pest *Bemisia tabaci*, their relatedness to the widespread greenhouse biological control agent *Encarsia formosa*, and because they harbor a bacterial endosymbiont capable of host reproductive manipulation, *Cardinium hertigii*. *Cardinium*, from the bacterial phylum *Bacteroidota*, shows independent evolution of reproductive manipulation from the well-known alphaproteobacterial *Wolbachia* [1]. Like other Hymenoptera, *E. suzannae* are haplodiploid and reproduce via arrhenotoky (arrhenotokous parthenogenesis): haploid males are produced via unfertilized eggs, and females are derived from fertilized diploid eggs [2]. Most *Encarsia* species, including *E. suzannae*, are also autoparasitoids. Specifically, females develop inside and consume the nymphs of the sweet potato whitefly, *B. tabaci*; male wasps develop as hyperparasitoids, consuming the pupae of conspecific females or other aphelinid parasitoids of whiteflies. After consuming their host, both male and female *Encarsia* pupate in the whitefly cuticle and emerge as adults [3]. Thus, many *Encarsia* species are effective parasites of the whitefly species. The latter are widespread pests causing billions of dollars in crop losses yearly as they can damage plants while feeding and transmit more than 200 different plant viruses to many plant species [4, 5]. As a result, *Encarsia* species have been widely used as pest control agents to limit whitefly populations in field or greenhouse settings [6–8]. The unusual autoparasitic biology [9], sex allocation behavior, and host selection of these intriguing wasps have also been studied [10].

Like many insects, *Encarsia* may be infected with maternally-transmitted intracellular bacterial endosymbionts, such as *Wolbachia* and *Cardinium*, which influence their transmission by manipulating host reproduction [11] or oviposition behavior [12] to favor infected females. These manipulations may induce asexual reproduction via thelytokous parthenogenesis [13, 14] or a type of male reproductive sabotage called cytoplasmic incompatibility (CI) [15]. CI causes the offspring of infected males and uninfected females to die early during development; on the other hand, females infected with the same symbiont can successfully mate with infected or uninfected males. This sabotage proceeds via a two-step mechanism: the symbiont alters the male sperm with a fatal modification, then rescues the infected offspring from this fatal modification when present in the fertilized egg. Together, the modification and rescue steps of CI grant infected females a relative fitness advantage over uninfected females, driving the symbiont to high frequencies in host populations [11]. The role of endosymbionts in arthropod biology, evolution, and speciation has been the subject of intense study [16–18]. Much of this research has focused on symbiont-induced CI, given its potential role in insect speciation [19–21], its application in arthropod pest population control [22, 23], and its ability to drive desirable genetic traits through populations (e.g., the resistance to arthropod-borne diseases) [24].

The *c*Eper1 strain of *Cardinium hertigii* is the causal agent of CI in *E. suzannae* [15]. This symbiosis between *c*Eper1 and *E. suzannae* is the best-studied instance of *Cardinium*-induced CI, and this strain of *Cardinium* has been well-characterized by genomic and transcriptomic data [1, 3]. However, sequence information of the host, *E. suzannae*, is extremely sparse: this species currently lacks a sequenced genome and a transcriptomic profile, hampering the molecular identification of host-symbiont interactions.

Here, we generated separate *de novo* assembled transcriptomes for male and female *E. suzannae* using previously obtained RNA-seq data that was generated to characterize the *Cardinium hertigii* transcriptome [3]. To our knowledge, there is only one other publicly available *Encarsia* transcriptome: that of the widely used greenhouse whitefly biocontrol agent *Encarsia formosa*, which was published as part of a phylogenetic characterization of Chalcidoidea parasitoid wasps [25, 26]. However, based on the morphology and lifestyle differences between *E. suzannae* and *E. formosa*, as well as their phylogenetic relationship, the two species are distantly related within the diverse *Encarsia* genus [27–30]. Our dataset will be a valuable asset for an ecologically important lineage within the chalcidoid wasps (Aphelinidae) that is sorely lacking sequencing data. We also provide the first molecular characterization of the host in the model *Cardinium* CI system.

## Methods

### Sample information and sequencing

We used the transcriptome data obtained by Mann *et al.* [3]. Whereas they focused on *Cardinium* data, here, we focused on the host (non-*Cardinium*) reads of the same dataset. The data was collected as described in the original manuscript [3]. In brief, the initial *E. suzannae* (NCBI:txid1892410) culture was obtained in 2006 in Weslaco, TX, from whitefly (*B. tabaci*) hosts. Male and female wasps were reared separately in a laboratory culture as described previously [3]. For females, mated *E. suzannae* were introduced to cages bearing whitefly nymphs on cowpea (*Vigna unguiculata*) plants. For males, unmated *E. suzannae* were provided with *Eretmocerus* sp. nr. *emiratus* larvae or pupae developing within whitefly nymphs. The total RNA from 6 groups of 350–500 male or female 1- to 3-day old *E. suzannae* wasps was extracted using TRIzol (Invitrogen). Next, the digestion of genomic DNA was done with the Turbo DNA-free kit (Ambion). The quality of the extracted RNA was assessed with a 2100 bioanalyzer (Agilent Technologies, RRID:SCR_018043). Three libraries for each sex were generated with the NEBNext Ultra RNA Library Prep Kit (Illumina) combined with the Ribo-Zero Magnetic Gold Kit (Epicentre Biotechnologies) for rRNA depletion. Samples were sequenced on an HiSeq 2500 platform (Illumina, RRID:SCR_016383) at the Vienna BioCenter Core Facilities (VBCF) NGS unit [31], producing a range of 127 to 162 million 50 bp paired-end reads per sample [3].

### Read preparation and assembly

Raw read files were processed with BBDuk (RRID:SCR_016969) from the BBTools software suite (v37.36, RRID:SCR_016968) [32] to remove the Illumina adapter sequences, trim and/or filter out whole reads with a quality score less than 15, and remove reads shorter than 36 bp after trimming using the following options: “ref=adapters.fa ktrim=r ordered k=23 hdist=1 mink=11 tpe tbo maq=15 qtrim=rl trimq=15 minlen=36”. We utilized FastQC (v0.11.9, RRID:SCR_014583) to visualize the sequence quality of each sample before and after trimming and to confirm the successful removal of adapter sequences [33]. Due to the complex biology of this species and its host insects, sequence contamination from a variety of organisms throughout the rearing system is inevitable, including *Cardinium c*Eper1, the different insect hosts of male and female *E. suzannae*, and the endosymbionts of those insect hosts. Thus, we employed a mapping-and-removal approach to enrich for *E. suzannae* reads prior to assembly and limit the generation of contaminating transcripts. For this approach, BBMap (RRID:SCR_016965) from BBTools was used to initially map the quality-trimmed reads to the genomes of *Cardinium hertigii c*Eper1 and the endosymbionts of *Bemisia tabaci* MEAM1, with which *E. suzannae* females and males have direct or indirect contact (i.e., *Hamiltonella defensa*, *Portiera aleyrodidarum*, and *Rickettsia* sp. MEAM1 [34, 35]). It was also determined that the *E.* sp. nr. *emiratus* hosts of *E. suzannae* males contain *Wolbachia* [36]; thus, the *Wolbachia* wPip genome was added and mapped to the male samples. Reads that did not map to any of these bacterial genomes with a greater than 94% identity were retained (to allow for a difference of 3 nucleotides between sequenced transcripts and reference endosymbiont genomes). These reads were then subsequently mapped to the *B. tabaci* MEAM1 genome with a more stringent 97% identity threshold using BBMap to avoid mapping *E. suzannae* reads from genes highly conserved in both *Encarsia* and *Bemisia* (see Table 1 for mapping and removal details). Again, only unmapped reads were retained for assembly, as these final reads were expected to be mainly attributed to *E. suzannae*.

**Table 1:** Pre-assembly contaminant read mapping and removal of *Encarsia suzannae* transcriptome sequencing data.

Organism	Reason for removal	Proportion of mapped trimmed reads	GenBank accession number
*Cardinium hertigii c*Eper1	CI-causing secondary *E. suzannae* endosymbiont	Female: 1.183% Male: 0.991%	GCA_000304455.1
*Portiera aleyrodidarum* MEAM1	Primary endosymbiont of *B. tabaci*	Female: 0.043% Male: 0.035%	GCA_002285875.1
*Rickettsia* sp. MEAM1	Secondary endosymbiont of *B. tabaci*	Female: 0.058% Male: 0.065%	GCA_002285905.1
*Hamiltonella defensa* MEAM1	Secondary endosymbiont of *B. tabaci*	Female: 0.040% Male: 0.037%	GCA_002285855.1
*Bemisia tabaci* MEAM1	Parasitized by female *E. suzannae* offspring and *E.* sp. nr. *emiratus*	Female: 5.343% Male: 5.289%	GCA_001854935.1
*Wolbachia pipientis* wPip (male only)	Secondary endosymbiont of *E.* sp. nr. *emiratus*, which is parasitized by male *E. suzannae* offspring	Female: N/A Male: 0.050%	GCA_000073005.1

List of the organisms whose reads were removed prior to assembly with Trinity. Quality-controlled reads were mapped to the genomes of the listed organisms, and the reads mapped to any of the references were removed.

We assembled separate transcriptomes for male and female *E. suzannae* whole adult wasps with the remaining unmapped reads using Trinity (v2.6.6, RRID:SCR_013048) and its default settings [37]. Transcript abundance was then estimated for each transcriptome with kallisto (RRID:SCR_016582) using the “align_and_estimate_abundance.pl” command bundled with Trinity [38]. Transcripts with an estimated abundance below 0.5 transcripts per million were removed from both assemblies as these may be lowly expressed isoforms of other transcripts, poorly assembled or chimeric transcripts, or simply contaminants and, thus, not from *Encarsia* [39, 40]. Next, TransDecoder (v5.5.0, RRID:SCR_017647) [41] was used to predict coding sequences within the remaining transcripts in each assembly and translate those coding sequences into predicted protein sequences with a minimum amino acid length of 67. Similar protein-coding sequences were then clustered using CD-HIT (v4.6.8, RRID:SCR_007105) [42, 43] with a 95% amino acid identity threshold, and the longest protein isoform of each cluster was selected as the representative sequence for that cluster. The final assemblies are presented as the nucleotide sequences of the representative proteins of each cluster. For a comprehensive list of the number of reads or transcripts at each step in the pipeline, see Table 2.

**Table 2:** *E. suzannae* transcriptome read and transcript statistics.

	*E. suzannae* female	*E. suzannae* male	*E. formosa*
**Total number of reads**	439,763,386	449,368,298	14,341,314
**Reads after trimming and mapping**	401,213,202	412,945,938	N/A
**Initial transcripts**	146,798	211,544	48,232
**Final transcripts**	122,465	136,359	47,852^∗ ^
**Coding sequences**	45,986^∗^	54,762^∗ ^	27,161
**Average length of final sequences (bp)**	697.74	692.03	772.51
**Assembly N50**	1,275	1,200	1,237
**Average% GC**	44.94	44.88	37.5
**% Annotated**	58.27	65.34	0
**Assembly software**	Trinity v2.6.6	Trinity 2.6.6	SOAPdenovo-Trans-31kmer (v1.01, RRID:SCR_013268)
**Reference**	This study	This study	[25]

Assembly and annotation statistics at each step in the pipeline for our *E. suzannae* transcriptomes compared to the previously-published *E. formosa* transcriptome assembly. The ^∗^ highlight the number and type of final sequences in the public version of each assembly.

### Quality control and data validation

Along with our mapping-and-removal approach to limit contaminations while enriching for *Encarsia* reads prior to assembly, we also utilized additional methods to improve the quality of our assemblies. First, to comply with the National Center for Biotechnology Information (NCBI)’s Transcriptome Shotgun Assembly (TSA) database requirements, we removed all coding sequences below 200 bp. Furthermore, we used blastn (RRID:SCR_001598) with the remaining sequences against the NCBI’s vector database to identify contaminating sequences and synthetic RNA spike-in controls; hits with a 100% nucleotide identity to vector sequences were removed from each assembly [44]. Prior to submission, any remaining coding sequences flagged by NCBI’s contamination check as sequencing vectors or contaminants were also removed. In total, 71 and 109 contaminating sequences were removed from the female and male assemblies, respectively.

The final assemblies were then assessed for completeness using Benchmarking Universal Single-Copy Orthologs (BUSCO) (v5.3.2, RRID:SCR_015008) in protein mode against the hymenoptera_odb10 reference lineage (v2020-08-05) [45, 46]. The female and male assemblies were found to possess, respectively, 82.1% and 82.6% of the 5,991 complete orthologs identified as single-copy and nearly universal within the order Hymenoptera (present in >90% of the tested species). This indicates a high level of completeness for both *E. suzannae* transcriptomes, although with varying degrees of duplication (shown in Table 3).

**Table 3:** Prediction of *E. suzannae* transcriptome assembly completeness using BUSCO.

	Male *E. suzannae*		Female *E. suzannae*	
BUSCO results	BUSCOs present	Percent of total	BUSCOs present	Percent of total
Complete BUSCOs	4,953	82.6%	4,915	82.1%
Complete single-copy BUSCOs	3,591	59.9%	4,492	75.0%
Complete duplicated BUSCOs	1,362	22.7%	423	7.1%
Fragmented BUSCOs	279	4.7%	280	4.7%
Missing BUSCOs	759	12.7%	796	13.2%
Total BUSCO groups searched	5,991	100%	5,991	100%

Assessment of assembly completeness using BUSCO v5.3.2 to search the assembled proteins against a database of proteins identified as Hymenopteran BUSCOs. All BUSCO groups searched were determined to be present in a single copy in >90% of the Hymenopteran species tested; therefore, a high number of complete single-copy BUSCOs indicates a comprehensive and non-redundant assembly [45].

One issue we could not rectify with the currently available sequencing data was the presence of *E.* sp. nr. *emiratus* transcripts within the male *E. suzannae* assembly. As mentioned above, haploid male *E. suzannae* eggs are laid into *Eretmocerus* pupae. Since this host does not have a sequenced genome (in contrast to *B. tabaci*), we could not apply the same mapping-and-removal approach to *E.* sp. nr. *emiratus*. This fact may at least partly explain the high number of total sequences and duplicated BUSCOs in the *E. suzannae* male assembly compared to the female one (see Tables 2 and 3). However, there are likely other contributing factors. Due to the relatedness of *Encarsia* and *Eretmocerus*, we could not differentiate sequences originating from either of these organisms at the read or assembled transcript level without their reference genomes. However, we are confident that the abundance of the *Eretmocerus* transcripts in the male assembly is low, and many may have been removed from the assembly during the transcript abundance filtering step. This is evidenced by the very low *Eretmocerus* biomass in/on fully emerged adult *E. suzannae* (larval *Encarsia suzannae* void their guts before pupation [47]). Additionally, using the average abundance of the *B. tabaci* reads as a proxy for the *E.* sp. nr. *emiratus* reads suggests an abundance of around 5% for *Eretmocerus* in either assembly (Table 1).

### Annotation

The male and female *E. suzannae* assemblies are available as unannotated coding sequences in the NCBI’s TSA database under the accession numbers GJLB00000000 and GJLI00000000, respectively. Here, we also provide the annotation information for both assemblies from multiple sources.

The final clustered proteins were annotated through the eggNOG-mapper (v2, RRID:SCR_021165) web-based pipeline using default settings to assign taxonomy information to the sequences and generate an annotation report including Gene Ontology terms, Pfam domains, KEGG (Kyoto Encyclopedia of Genes and Genomes) pathways, and other relevant information [48, 49]. Additionally, the final proteins were searched against the NCBI’s non-redundant (nr) protein database (release 242.0) using DIAMOND (RRID:SCR_016071) and the “–very-sensitive” option [50]. The final proteins were also searched using blastp (RRID:SCR_001010) [51, 52] and an *e*-value cutoff of 10^−5^ against a targeted database of well-annotated predicted insect proteomes: *Nasonia vitripennis* Nvit_psr_1_1 (Genbank accession: GCA_009193385.2), *Trichogramma pretiosum* Tpre_2_0 (Genbank accession: GCA_000599845.3), and *Bemisia tabaci* MEAM1 (Genbank accession: GCA_001854935.1). Although not closely related to *Encarsia*, *Bemisia* was included in the targeted insect database. Its thorough annotation and presence as an outgroup may be useful in annotating proteins retained in *Encarsia* that *Nasonia* or *Trichogramma* may have lost. This database was also found to generate fewer hits labeled as “hypothetical” or “uncharacterized” when compared to a search against the nr protein database. The annotation results from each reference for both assemblies were pooled into a single Microsoft Excel spreadsheet (Additional File 1). We also provide a .fasta file for each assembly containing the final nucleotide sequences and the sequence headers containing the annotations from blastp against the targeted insect database (female: Additional file 2; male: Additional file 3).

Approximately 58% and 65% of the female and male assembled proteins were annotated using one of the listed methods, with the characterization against the NCBI’s nr database annotating the highest number of proteins (26,155 female and 35,073 male), followed closely by the targeted insect database (24,478 female and 33,353 male). Some transcripts of note that were annotated in both the male and female assemblies are putative homologs to an array of insect sex-determination genes characterized in *Drosophila*. These homologs included *sex lethal* (*sxl*), the master regulator of the *Drosophila* sex-determination cascade, and some genes it regulates, including *transformer* (*tra*), *doublesex* (*dsx*), and *fruitless* (*fru*). *Sex lethal* controls the splicing of *tra*, which is involved in the sex-specific splicing of *dsx* and *fru* [53] and results in either male isoforms of *dsx* and *fru* or a female isoform of *dsx* and a truncated and untranslated female *fru* isoform. The different *dsx* isoforms are crucial for male and female somatic sexual development, while *fru* appears to be key in the male courtship behavior of *Drosophila* [54, 55]. We also searched the assemblies for homologs of *wasp overruler of masculinization* (*wom*) [56] but found none. This gene was identified in *N. vitripennis* as the instructor of sex determination via the activation of *tra* expression and autoregulation, which results in female development. However, we cannot rule out the presence of *wom* in *E. suzannae* as this gene in *N. vitripennis* is mainly transcribed in diploid (female) embryos prior to 7 h post oviposition and is not expressed in adults, which we sampled for our transcriptome assemblies. We also did not find homologs of *complementary sex determiner* (*csd*), the instructor of sex determination in *Apis mellifera*.

Sex determination in the Chalcidoidea has been a matter of some speculation [57]. However, the presence or absence of these transcripts provides insights into the nature of sex determination and development in *E. suzannae* and lays the foundation for understanding how the mechanisms of sexual development in *Encarsia* may interface with the reproductive manipulation of *Cardinium*. Particularly applicable are cases of symbiont-induced parthenogenesis, in which unfertilized eggs are diploidized by the endosymbiont and biological females are produced [13, 58].

Furthermore, the identification of many transcripts harboring coding sequences annotated as putative venom proteins in both the male and female *E. suzannae* transcriptomes is notable as these are believed to be important mechanisms used by female parasitoid wasps to enhance the survivability of their offspring. Venom proteins are diverse and predicted to have a variety of impacts on the host undergoing parasitism, including immune system suppression, developmental arrest, lipid accumulation, and apoptosis [59]. In the case of *E. suzannae*, parasitism causes the whitefly host to undergo developmental arrest during a late nymphal stage. As arrest occurs regardless of wasp larva survival, it is possible that it is induced by venom injected into the whitefly during oviposition [15]. The presence of predicted proteins annotated as venom proteins in the male *E. suzannae* assembly is intriguing since only female wasps host feed and lay eggs into their host, while adult males seemingly have no need to express venom genes. It is unclear whether these putative proteins are actually venom genes expressed in male *E. suzannae* or if they were annotated as such due to the presence of domains similar to those found in venom proteins. Regardless, detecting putative venom proteins in *E. suzannae* provides more insight into how these wasps effectively parasitize their hosts. However, it should be noted that reliable identifications of venom proteins require additional experimental verifications.

### Transcriptome comparisons

As stated above, the only other publicly available transcriptome of an *Encarsia* species belongs to *E. formosa* [26]; thus, limited comparisons can be made within this genus. An overview of all currently known *Encarsia* transcriptomes is shown in Table 2. Compared to the *E. formosa* transcriptome assembly, the male and female *E. suzannae* assemblies were generated from more initial reads and produced more pre-filtering transcripts, meaning they could be subject to more stringent transcript filtering than the *E. formosa* assembly. While the *E. formosa* assembly underwent limited post-assembly contaminant filtering, the *E. suzannae* assemblies utilized additional measures to (1) limit potential nonsense, low-abundance, and redundant transcripts through post-assembly filtering and processing, and (2) eliminate as many contaminants as possible prior to the assembly via mapping-and-removal. Furthermore, the publicly available *E. formosa* assembly consists of full-length mRNA transcripts instead of coding sequences, as seen in the *E. suzannae* assemblies [25]. After running TransDecoder on the *E. formosa* transcripts, only 27,161 coding sequences were predicted using a minimum length of 50 amino acids. This indicates that the female (45,986) and male (54,762) *E. suzannae* assemblies contain twice or nearly twice as many coding sequences compared to the *E. formosa* assembly, even though the *E. formosa* coding sequences were predicted with a shorter minimum protein size than *E. suzannae*.

**Figure 1.**
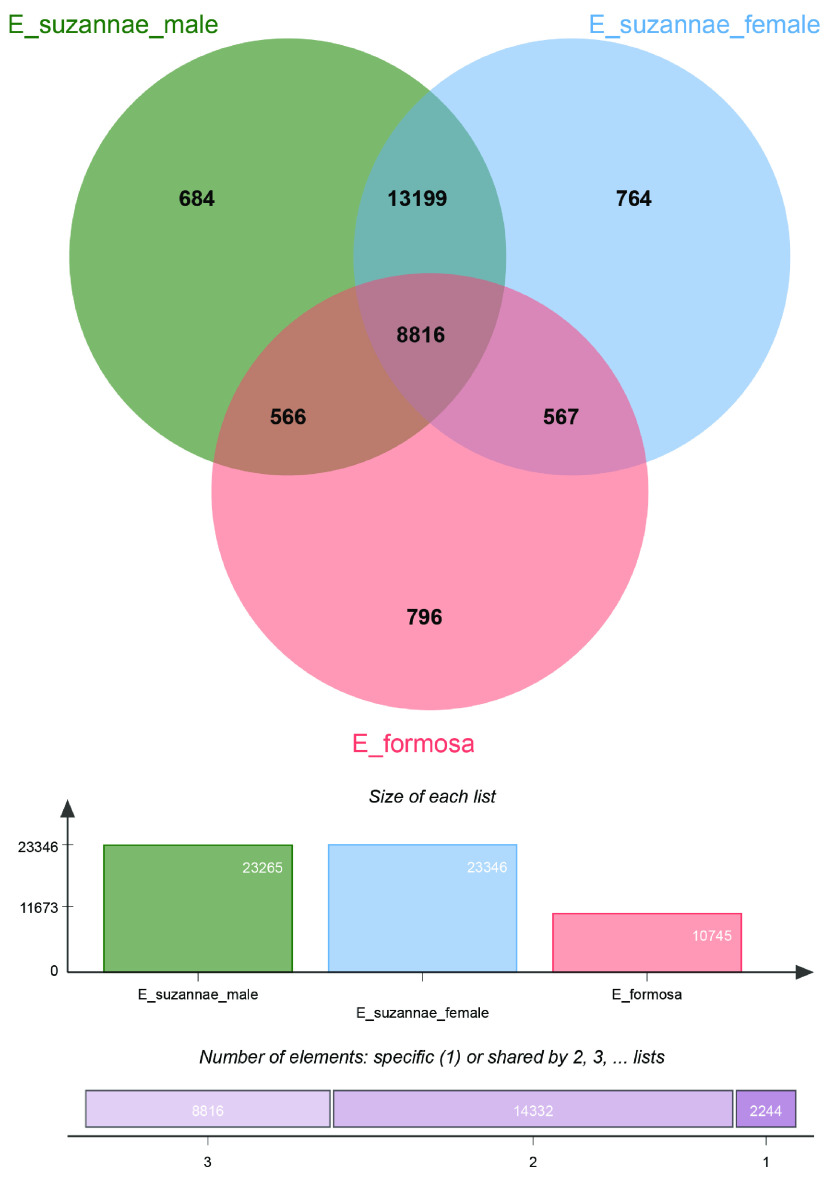
Orthologous groups between the *E. formosa* females and the male and female *E. suzannae* transcriptomes. The above figure shows an OrthoVenn2 diagram of the orthologous groups between the *E. formosa* females and the male and female *E. suzannae* (*e*-value = 1 × 10^−5^) [60]. TransDecoder, using a minimum amino acid length of 50, was run on the *E. formosa* assemblies to obtain the coding sequences. The resulting peptide sequence output (27,161 sequences) was tested against the predicted proteins from the male and female *E. suzannae* transcriptomes. The top Venn diagram depicts the number of orthologous protein clusters shared between the three transcriptomes. The middle bar graph depicts the total number of orthologous clusters present for each transcriptome. Lastly, the bottom graph shows (left to right) the number of clusters that were shared by all three transcriptomes, by any two transcriptomes, or were unique to one of the three assemblies.

Finally, OrthoVenn2 (RRID:SCR_022504) was used to determine the orthologous groups between the predicted proteins in both the *E. suzannae* assemblies presented in this paper and the *E. formosa* assembly published elsewhere [26, 60]. Using the default settings and an *e*-value cutoff of 1 × 10^−5^, 8816 orthologs were found to be shared across all three transcriptomes, and a total of 22,015 orthologous groups were shared between male and female *E. suzannae* out of the total of 23,265 and 23,346 clusters, respectively (see Figure 1). These results indicate a high degree of similarity between the different sex assemblies while showing the presence of over one thousand sex-specific protein clusters. It is also striking that the female and male *E. suzannae* transcriptomes are equally similar to the *E. formosa* transcriptome, although *E. formosa* exists as an asexual species consisting of nearly all females (due to the presence of parthenogenesis-inducing *Wolbachia*), and its transcriptome therefore only reflects female individuals [61].

## Conclusion and re-use potential

**Table 4:** Software and version specifications.

Software	Usage	Version	Reference(s)
BBTools	BBDuk for read trimming; BBMap for read mapping	37.36	[32]
FastQC	Visualization of sequence quality	0.11.9	[33]
SAMtools (RRID:SCR_005227)	.bam file manipulation	1.10	[62]
Trinity	*De novo* transcriptome assembly	2.6.6	[37]
kallisto	Transcript abundance estimation	0.46.2	[38]
TransDecoder	Prediction of coding sequences	5.5.0	[41]
CD-HIT	Clustering similar protein sequences	4.6.8	[42, 43]
BUSCO	Assessing assembly completeness	5.3.2	[45]
eggNOG-mapper	Annotation of assembled proteins	2.1.6	[48, 49]
Blast+	Annotation of assembled proteins	2.11.0	[51]
Diamond	Annotation of assembled proteins	2.0.4	[50]
OrthoVenn2	Orthologous protein group clustering and visualization	N/A	[60]

We are confident that our assemblies are among the purest possible transcriptome representations of *E. suzannae* that can be obtained with the currently available data and tools for assembly and filtering (for a list of all software names and versions utilized in this study, see Table 4). This study is also one of the first to present sex-specific transcriptome assemblies of a single insect species. In an organism such as *E. suzannae* – where males and females develop within different hosts, are impacted differently by endosymbiotic bacteria, and exhibit distinct behaviors – it is highly valuable to have the availability of a reference database for both sexes to ensure more accurate studies when wasps of only one sex are used. Furthermore, these assemblies greatly expand our host knowledge of the *Cardinium c*Eper1 CI system and pave the way for future studies exploring how this endosymbiont interacts with its *E. suzannae* host in causing CI. We also believe that these data will be a valuable reference when studying the diverse members of the ecologically important genus *Encarsia* and other chalcidoid parasitic wasps, many of which have interesting biology and potential as pest biological control agents.

## Data Availability

All the raw sequencing data and the final assemblies from this study are publicly available. The *E. suzannae* female and male raw read data and unannotated assemblies were submitted to the NCBI’s Sequence Read Archive (SRA) and Transcriptome Shotgun Assembly (TSA) databases under the BioProjects PRJNA737477 for male *E. suzannae* and PRJNA737478 for female *E. suzannae*. Detailed annotation information from multiple sources is provided alongside the annotated female and male assemblies in FASTA format in the GigaDB repository [63].

## List of abbreviations

BUSCO: Benchmarking Universal Single-Copy Orthologs; CI: Cytoplasmic incompatibility; KEGG: Kyoto Encyclopedia of Genes and Genomes; NCBI: National Center for Biotechnology Information; nr: non-redundant; SRA: Sequence Read Archive; TSA: Transcriptome Shotgun Assembly; VBCF: Vienna BioCenter Core Facilities.
