# Supplementary material for: Sex-based de novo transcriptome assemblies of the parasitoid wasp Encarsia suzannae, a host of the manipulative heritable symbiont Cardinium hertigii
Source: GigaByte. 2022 Sep 2;2022:gigabyte68. doi: 10.46471/gigabyte.68 (PMC9693781; doi:10.46471/gigabyte.68)
Supplement: Peer review history

# Reviewer report: Review MS


Reviewer name and names of any other individual's who aided in reviewer Zhizhi WangDo you understand and agree to our policy of having open and named reviews, and having your review included with the published papers. (If no, please inform the editor that you cannot review this manuscript.)YesIs the language of sufficient quality?YesPlease add additional comments on language quality to clarify if needed
Are all data available and do they match the descriptions in the paper? NoAdditional CommentsAre the data and metadata consistent with relevant minimum information or reporting standards? See GigaDB checklists for examples &lt;a href="http://gigadb.org/site/guide" target="_blank"&gt;http://gigadb.org/site/guide&lt;/a&gt;YesAdditional CommentsIs the data acquisition clear, complete and methodologically sound?YesAdditional CommentsIs there sufficient detail in the methods and data-processing steps to allow reproduction?YesAdditional CommentsIs there sufficient data validation and statistical analyses of data quality? NoAdditional CommentsIs the validation suitable for this type of data?YesAdditional CommentsIs there sufficient information for others to reuse this dataset or integrate it with other data?YesAdditional CommentsAny Additional Overall Comments to the AuthorRecommendationMajor Revision


# Reviewer report: Review MS


Reviewer name and names of any other individual's who aided in reviewer NoDo you understand and agree to our policy of having open and named reviews, and having your review included with the published papers. (If no, please inform the editor that you cannot review this manuscript.)YesIs the language of sufficient quality?YesPlease add additional comments on language quality to clarify if needed
Are all data available and do they match the descriptions in the paper? YesAdditional CommentsAre the data and metadata consistent with relevant minimum information or reporting standards? See GigaDB checklists for examples &lt;a href="http://gigadb.org/site/guide" target="_blank"&gt;http://gigadb.org/site/guide&lt;/a&gt;YesAdditional CommentsIs the data acquisition clear, complete and methodologically sound?YesAdditional CommentsIs there sufficient detail in the methods and data-processing steps to allow reproduction?YesAdditional CommentsIs there sufficient data validation and statistical analyses of data quality? YesAdditional CommentsEven though the data presented are not very informative, there is no any obvious flaw throughout the MS. Is the validation suitable for this type of data?YesAdditional CommentsIs there sufficient information for others to reuse this dataset or integrate it with other data?YesAdditional CommentsAny Additional Overall Comments to the AuthorRecommendationMinor Revision


# Reviewer report: Re-Review MS


Comments on revised manuscriptThe authors have addressed most of my concerns, so I would like to recommend the paper for publication.


# Editor report: Accept


Comments to the AuthorPlease check the proofs.
